# Effects of caffeine and tannin treatments on biodegradation resistance and fire retardancy of oriented strand board (OSB) panels

**DOI:** 10.1617/s11527-026-03128-y

**Published:** 2026-05-11

**Authors:** Yeray Manuel López-Gómez, Flore Anna Salomé-Simon, Aitor Barbero-López, Alain Cloutier, Antti Haapala

**Affiliations:** 1 Department of Chemistry, University of Eastern Finland, P.O. Box 111, 80101 Joensuu, Finland; 2 Renewable Materials Research Center, Université Laval, Québec, Canada; 3 FSCN Research Centre, Mid Sweden University, 85170 Sundsvall, Sweden

**Keywords:** Biodegradation, OSB panels, Wood preservation, Fire resistance

## Abstract

This study investigates the effectiveness of caffeine and tannin as alternative preservatives for oriented strand board (OSB) panels, focusing on their leaching, fungal decay resistance, and fire performance. Untreated, caffeine-treated, tannin-treated, and industrial reference panels were subjected to standard tests of these performance features. The leaching results indicated that the caffeine-treated panels retained a similar level of chemical content and exhibited lower mass loss after leaching compared to the tannin-treated panels, suggesting a higher concentration of caffeine remained in the panels following the leaching test. Both caffeine and tannin treatments significantly reduced fungal decay caused by *T. versicolor*, *C. puteana*, and *G. trabeum* compared to untreated and industrial reference panels, with caffeine-treated panels demonstrating superior protection among all tested treatments. Fire resistance tests showed that most of the leached panels exhibited longer times to ignition than non-leached panels. However, non-leached panels had higher peak heat release rates. The study concludes that caffeine and tannin treatments effectively protect against fungal decay, but their impact on fire resistance remains inconclusive, with no clear enhancement. These findings highlight the potential of caffeine and tannin as natural wood preservatives, particularly in conditions where decay is a primary concern, though further research is needed to optimize their performance under various environmental conditions.

## Introduction

The construction industry increasingly prioritizes sustainable methods and materials to mitigate environmental impact. Prominent among these materials are wood-based products such as plywood, glue-laminated timber (GLT), cross-laminated timber (CLT), particleboards, and oriented strandboards (OSB). These materials significantly contribute to sustainable construction due to their renewable nature and lower carbon footprint than traditional materials like steel and concrete. However, there is potential to further enhance their properties and environmental benefits through advanced manufacturing techniques and innovative applications.

Numerous studies have focused on developing more sustainable panels to reduce environmental impact [1]. For instance, Mansouri et al. [2] and Navarrete et al. [3] utilized a natural-based adhesive composed of low molecular mass lignin and tannins to replace synthetic resins. Similarly, Wang et al. [4] demonstrated the application of whey protein-based water-resistant adhesives for plywood, and Zhao et al. [5] reported the efficiency of biomass-based formaldehyde-free bio-resin for wood panel production. Other studies have explored using agricultural and forest residues, such as straw and tomato stalk [6], for panel production.

In addition to enhancing sustainability, research has also focused on improving the material properties of wood-based panels. This includes the addition of phase change material for efficient thermal energy storage [7], utilizing rice straw in wood particle composite boards for sound absorption [8], enhancing fire resistance by applying separate solutions of phytic acid and sodium silicate to wood particles [9], developing particleboard with improved mechanical and physical properties using nanoparticles [10], and improving the dimensional stability of OSB panel through thermal post-treatment [11, 12]. Despite these efforts, there is a clear lack of research on potential treatments to improve the properties of wood-based panels, including their durability.

Wood-based products are susceptible to biodegradation by decay fungi when exposed to moisture. To enhance wood decay resistance, various treatments and modifications have been developed over time [13, 14]. While some treatments, such as creosotes [15] and chromated copper arsenate [16], are restricted due to strict legislation and environmental concerns, alternative treatments are currently being used as replacements. These alternatives include ammoniacal copper citrate (CC), copper azole (CA), alkaline copper quaternary (ACQ) and copper dimethyl-dithio-carbamate (CDDC), [17, 18]. Nevertheless, these treatments do not offer a permanent solution to wood preservation.

Current studies in wood protection are focusing on developing bio-based wood preservatives such as coffee waste extracts [19], monoterpenes [20], propolis extracts, mistletoe extractives [21], tannins [22, 23], and hydrothermal liquefaction liquids from spent mushroom and tomato residues [24].

Various methods have been reported to improve the decay resistance of wood panels. Thermal modification has been shown to enhance decay resistance [25]. Additionally, biological treatments, such as using *Gliocladium roseum* to protect hardwood logs destined for panel manufacturing, have been explored [26]. This treatment has also been used to improve the mold resistance of aspen strands [27]. Previous studies have also evaluated the decay resistance of bamboo-oriented strand boards treated with copper-based preservatives [28]. Cai et al. [29] investigated the use of a β-cyclodextrin-allyl isothiocyanate complex as a natural preservative for OSB panels. Furthermore, the effects of copper-based preservative technologies on the resistance of aspen strand boards to biological degradation have been examined [30].

Caffeine and tannins have demonstrated significant biocidal efficacy against wood-degrading fungi, insects, and molds, highlighting their potential as environmentally friendly wood protection agents [31, 32]. Their biocidal activity has been attributed to different mechanisms, including interference with fungal metabolism in the case of caffeine and the ability of tannins to complex proteins and metal ions, thereby inhibiting microbial growth.

These characteristics have positioned both compounds as promising alternatives to conventional synthetic wood preservatives.

Despite their effectiveness, a major limitation for the practical application of caffeine- and tannin-based treatments is their high-water solubility, which results in pronounced leachability when applied to wood exposed to moisture. This drawback significantly reduces long-term durability and restricts their use in outdoor or high-humidity environments. Consequently, recent research efforts have focused on developing strategies to improve the fixation of these bio-based compounds within wood substrates and to reduce their susceptibility to leaching.

Several approaches have been proposed to address this issue, including the incorporation of crosslinkers [33], the use of aminosilanes to promote chemical bonding with wood components [34], and the application of geopolymer-based systems to enhance physical encapsulation [35]. Additional strategies such as thermal modification [36] and oil treatments [37] have also been investigated, aiming to reduce wood hygroscopicity and thereby limit the mobility of water-soluble preservatives. These studies demonstrate that improving fixation is a critical factor for translating the intrinsic biocidal properties of caffeine and tannins into effective and durable wood protection systems.

Beyond biological resistance, tannin-based compounds have attracted further interest due to their multifunctional potential. In particular, tannic acid has been investigated for its fire-retardant properties [38], expanding its applicability beyond conventional preservation. For example, formulations based on tannic acid combined with sodium hydroxide have been reported to provide effective flame-retardant coatings for cotton fabrics [39]. Such findings suggest that tannin-based systems may offer added functional benefits when applied to lignocellulosic materials, including wood, and underline the relevance of further research into multifunctional, bio-based wood protection strategies.

Utilizing these biobased preservatives for OSB panels offers a promising approach to enhance wood decay resistance and minimize leaching, attributed to the integration of phenol–formaldehyde during panel manufacturing, which serves as a cross-linking agent.

These advancements underscore the potential of wood-based materials in achieving more sustainable and efficient construction practices. The ongoing research and development in this field aim to optimize both the environmental and functional attributes of these materials, reinforcing their role in the future of sustainable construction. The antifungal performance observed in the present study is consistent with previous reports on tannin-based preservatives, which showed comparable inhibition levels against tested fungi.

The objective of this study is to evaluate and compare the impacts of tannin and caffeine-based wood preservatives on the leachability, biodegradation resistance, and fire retardancy of OSB panels.

## Material and methods

### Materials and strands impregnation

Aspen (*Populus tremuloides*) strands were sourced from Arbec Forest Products Inc. (Shawinigan, Quebec, Canada). The strands were impregnated with solutions of either caffeine or tannin, each diluted to a 1% concentration in distilled water. Caffeine (C_8_H_10_N_4_O_2_, 99% purity) was supplied from Sigma-Aldrich, while the tannin-rich, water-soluble extract Colatan GT 10, produced by Unitán in Argentina, was acquired from Haarla Ltd. (Tampere, Finland). Colatan GT 10 is derived from the bark of the Colorado Quebracho tree (*Schinopsis lorenzii*), using physicochemical extraction methods. Colatan GT 10 was selected owing to its substantial availability in commercially relevant quantities and its cost-effectiveness, which positions it as a practical candidate for extensive applications like wood preservation. Phenol formaldehyde liquid resin (70% solid content) and wax emulsion (59% solid content) were provided by Hexion Inc. (Levis, Quebec, Canada).

The impregnation of Aspen strands was carried out using the Bethell full-cell process. The procedure began with the application of a vacuum pressure of 15 kPa for 20 min at a temperature of 20 °C. Following the vacuum stage, the system was pressurized to 1000 kPa for 60 min, also at 20 °C. The chemical retention in the strands was calculated by comparing their oven-dried mass (at 103 ± 2 °C) before and after treatment.

### Panel manufacture

The strands were first screened to eliminate fine particles. They were oven-dried at 103 ± 2 °C to achieve a constant dry mass prior to chemical impregnation. Following impregnation using the Bethell full-cell process, the strands were subjected to an additional drying cycle to determine the post-treatment dry mass, allowing the calculation of chemical retention. The strands were subsequently conditioned to a target moisture content of 3% in preparation for panel manufacturing.

**Fig. 1:**
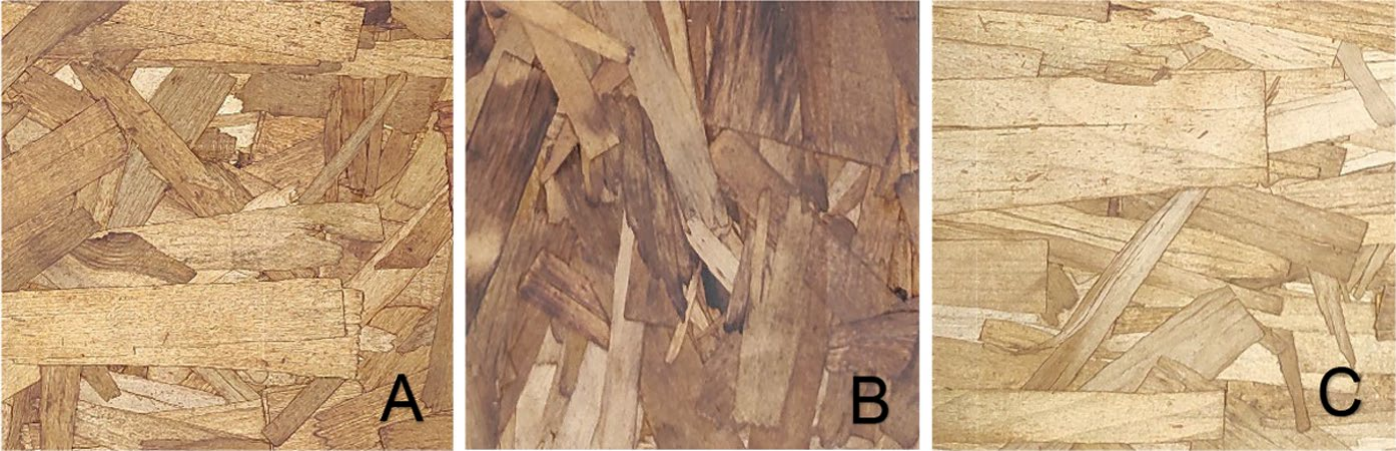
Visual comparison of untreated (**A**), tannin-treated (**B**), and caffeine-treated (**C**) OSB panels

The mass proportion of the surface/core/surface layers was 30:40:30. The phenol formaldehyde and wax content in the core layer were 5% and 0.5% of the oven-dry wood mass, respectively, whereas it was 7% and 1% in the surface layers. The strands were then homogeneously blended with liquid phenol formaldehyde resin and wax emulsion using an in-house made rotary drum blender. The mat was formed within a mold using a screening box to accurately align the strands in the intended direction. The orientation of the surface-layer strands was set perpendicular to the strands in the core layer. The mat was hot pressed at 190 °C during a pressing cycle of 390s, using a Dieffenbacher North America hot press (Windsor, ON, Canada) heated with thermal oil. The target density of the panel was 630 kg/m^3^, and the dimensions were 760 mm × 760 mm × 10 mm. A total of 11 panels were manufactured, representing different treatments: four untreated, three treated with caffeine, four treated with tannin, and four industrial reference panels from Arbec Forest Products Inc were used for comparison (Table 1). After hot pressing, the panels were stored in a conditioning room at 20 °C and 65% relative humidity to obtain an equilibrium moisture content (EMC) of about 8%. The panels were then cut to the necessary dimensions for further testing (Fig. 1).

**Table 1:** Number of panels produced for each treatment group

Panel	Number of replicates
Untreated	4
Tannin-treated	4
Caffeine-treated	3
Industrial reference	4

### Leaching test

Half of the specimens in each test were randomly selected to undergo a leaching procedure following the European standard EN 84 (CEN 1997) [40]. These specimens were submerged in Milli-Q water at a volume ratio of 5:1 (v/v) for 14 days. The water was replaced a total of nine times during this period. The first two changes occurred at 24 and 48h, with the remaining seven changes spread over the next 12 days, at intervals ranging from 24 to 72h. After the leaching process, the specimens were oven-dried at 103 ± 2 °C until a stable mass was reached. The dry mass before and after the leaching test was then measured and compared.

### Decay test

The decay test followed ASTM D2017- 05 standard [41]. The growth media were prepared by combining 2% malt extract and 1.5% agar in Milli-Q water, followed by autoclaving (120 °C, 20 min). Subsequently, 20 mL of liquid culture medium was dispensed into each bottle (450 mL) under sterile conditions.

In this experiment, 24 replicates of each panel (12 leached and 12 non-leached) of 25 mm × 25 mm × 10 mm in size were exposed to three different rot fungi: *Gloeophyllum trabeum* (strain ATCC 11539), *Coniophora puteana* (strain ATCC 36336), and *Trametes versicolor* (strain ATCC 12679), purchased from Cedarlane, 4410 Paletta Court, Burlington, Ontario, Canada, L7L 5R2. The inoculation was conducted under sterile conditions, with a plug (10 mm^2^) of actively growing fungus placed at the centre of each bottle. Following inoculation, the bottles were maintained in a growth chamber at 65 ± 5% relative humidity (RH) and a temperature of 22 ± 2 °C. In each bottle, two wood specimens from the same treatment—one leached and one non-leached—were exposed to the fungus once the mycelium had fully covered the entire surface of the culture medium. All wood specimens utilized in the experiment underwent autoclaving (120 °C for 20 min) and were maintained in a sterile condition within a laminar flow hood before being introduced into the bottles containing fungi. Additionally, ceramic supporters were placed inside each bottle to prevent direct contact between the wood and the growth medium. The bottles were placed in a growth chamber maintained at 25 ± 1 °C and 70 ± 2% relative humidity (RH) for 16 weeks. Later, the dry mass of the wood specimens was measured at 103 ± 2 °C and compared with their initial dry mass before exposure to decay, allowing the calculation of the mass loss (wt-%) attributed to the fungi.

### Fire resistance test

The fire resistance performance of the panels was evaluated through cone calorimeter testing, in accordance with ISO 5660-1 [42]. A total of four replicates were prepared and tested for each panel, two leached and two non-leached samples, to assess the potential influence of leaching exposure on fire behavior. The dimensions of each sample were standardized to 100 mm × 100 mm × 10 mm. All tests were conducted with the samples positioned horizontally, using a conical radiant electric heater to provide a heat flux of 50 kW/m^2^.

### Statistical analysis

Statistical analysis was performed using R software. For each evaluated property, the mean and standard error were determined. An analysis of variance (ANOVA) was then conducted, followed by Tukey’s range test for post-hoc comparison between treatments. Prior to performing ANOVA, assumptions of normality and homogeneity of variance were evaluated using Q–Q plots, the Shapiro–Wilk test (*p* > 0.05), and Levene’s test (*p* > 0.05). An analysis of variance (ANOVA) was then conducted, followed by Tukey’s range test for post-hoc comparison between treatments.

## Results

### Leaching

Table 2 shows the impact of leaching and chemical retention across different treatments of OSB panels. The untreated and industrial reference panels exhibited similar mass losses due to leaching, with values of 3.02 ± 0.35% and 3.27 ± 0.27%, respectively. Tannin-treated panels demonstrated a chemical retention of 3.06%, while caffeine-treated panels showed a chemical retention of 3.40%. The tannin-treated panels experienced a greater mass loss due to leaching (4.21 ± 0.05%) than the caffeine-treated panels (3.04 ± 0.12%).

**Table 2:** Dry retention values after impregnation treatment and mass loss due to leaching. Values are presented as mean ± standard error (n = 48)

Panel	Chemical retention after treatment (%)	Mass loss due to leaching (%)
Untreated	–	3.27 ± 0.27
Tannin-treated	3.06	4.21 ± 0.05
Caffeine-treated	3.40	3.04 ± 0.12
Industrial reference	–	3.02 ± 0.35

### Decay

The decay test results, presented in Table 3, revealed that untreated and industrial panels, both with and without leaching exposure, experienced the highest mass loss across all three fungi. In contrast, caffeine and tannin treatments significantly reduced mass loss compared to the control and industrial reference samples. For *T. versicolor*, caffeine-treated samples showed a mass loss of 3.3 ± 0.3% under leached conditions and 1.6 ± 0.3% under non-leached conditions. Tannin-treated samples exhibited 6.3 ± 0.4% mass loss when leached and 1.3 ± 0.2% when non-leached. When exposed to *C. puteana*, caffeine-treated samples had a mass loss of 7.2 ± 1.0% when leached and 4.4 ± 1.4% when non-leached, whereas tannin-treated samples showed 10.3 ± 0.1% and 3.3 ± 0.3% mass loss for leached and non-leached samples, respectively. For *G. trabeum*, caffeine-treated samples had mass losses of 5.2 ± 0.8% in leached and 2.0 ± 0.1% in non-leached samples, while tannin-treated samples showed a mass loss of 8.9 ± 1.5% in leached conditions and 3.0 ± 0.3% in non-leached conditions. Although both caffeine and tannin treatments resulted in higher mass loss after leaching, a statistically significant difference between leached and non-leached samples was only observed in tannin-treated samples when exposed to *T. versicolor* and *G. trabeum*. The mass loss caused by the three fungi was also significantly higher in untreated panels and industrial references than in leached caffeine-treated and tannin-treated samples, showing the effectiveness of the treatments even after leaching.

**Table 3:** Mass loss due to decay (%). Results are presented as mean ± standard error (*n* = 16). Different letters indicate significant differences between treatments based on Tukey’s range test

		Mass loss due to decay (%)		
Panel	Leaching exposure	*T. versicolor*	*C. puteana*	*G. trabeum*
Untreated	Leached	18.7 ± 2.5^a^	21.2 ± 2.1^a^	19.2 ± 2.2^a^
	Non-leached	18.5 ± 2.1^a^	20.2 ± 2.1^a^	17.0 ± 2.3^a^
Caffeine-treated	Leached	3.3 ± 0.3^bc^	7.2 ± 1.0^bc^	5.2 ± 0.8^bc^
	Non-leached	1.6 ± 0.3^c^	4.4 ± 1.4^c^	2.0 ± 0.1^c^
Tannin-treated	Leached	6.3 ± 0.4^b^	10.3 ± 0.1^bc^	8.9 ± 1.5^b^
	Non-leached	1.3 ± 0.2^c^	3.3 ± 0.3^c^	3.0 ± 0.3^c^
Industrial reference	Leached	16.6 ± 1.9^a^	18.5 ± 1.4^a^	22.5 ± 1.6^a^
	Non-leached	15.5 ± 2.9^a^	17.7 ± 1.3^a^	16.0 ± 1.4^a^

### Fire resistance

The fire resistance performance of OSB panels treated with caffeine, tannin, and industrial reference, along with the untreated control, was evaluated under leached and non-leached conditions (Table 4).

**Table 4:** Fire parameters measured during the fire test. Results are presented as mean ± standard error (n = 8). Different letters indicate significant differences between treatments according to Tukey’s range test. An asterisk (*) denotes specimens that underwent a leaching test

Panel	Time to ignition (s)	Mean heat release rate (kW/m^2^)	Peak heat release (kW/m^2^)	Mass loss (%)	Total smoke (m^2^/m^2^)	Time to flameout (s)	Total heat release (MJ/m^2^)
Untreated	*34.5 ± 1.2^a^	*97.8 ± 2.2^b^	*219.3 ± 5.8^bc^	*80.9 ± 0.3^ab^	*344.3 ± 13.1^c^	*795.3 ± 43.0^abc^	*84.2 ± 2.5^ab^
	30.6 ± 1.0^abc^	108.6 ± 3.1^ab^	255.9 ± 7.2^abc^	77.6 ± 1.5^c^	481.4 ± 20.7^ab^	688.0 ± 19.3^bc^	82.9 ± 3.7^b^
Caffeine-treated	*28.7 ± 3.2^abc^	*102.1 ± 3.2^ab^	*241.3 ± 10.5^abc^	*80.9 ± 0.5^ab^	*373.3 ± 29.1^bc^	*758.5 ± 23.8^abc^	*85.6 ± 4.0^ab^
	26.8 ± 1.7^abc^	102.3 ± 2.3^ab^	270.8 ± 8.3^a^	80.6 ± 0.4^ab^	476.8 ± 32.8^abc^	813.7 ± 52.4^abc^	91.2 ± 4.7^ab^
Tannin-treated	*34.3 ± 2.2^ab^	*103.3 ± 3.3^ab^	*236.5 ± 9.7^abc^	*79.2 ± 0.6^bc^	*371.8 ± 21.2^bc^	*828.9 ± 29.9^ab^	*91.4 ± 4.1^ab^
	26.4 ± 1.5^c^	111.6 ± 2.5^a^	252.1 ± 10.6^abc^	79.0 ± 0.2^bc^	405.3 ± 60.1^bc^	662.1 ± 23.0^c^	82.5 ± 2.4^b^
Industrial reference	*25.1 ± 1.3^c^	*102.9 ± 2.2^ab^	*210.4 ± 6.9^c^	*82.3 ± 0.3^a^	*476.7 ± 14.2^abc^	*872.8 ± 39.3^a^	*97.6 ± 2.7^a^
	26.6 ± 1.8^bc^	112.2 ± 3.6^a^	256.9 ± 18.9^ab^	80.0 ± 0.2^abc^	587.2 ± 22.2^a^	740.0 ± 29.0^abc^	91.2 ± 1.4^ab^

Time to ignition varied significantly across the treatments. The untreated leached panels exhibited the longest ignition time (34.5 ± 1.2s), which was significantly higher than the industrial reference leached panels and the tannin-treated non-leached panels, which had the shortest ignition time. The tannin-treated non-leached panels showed significantly lower ignition times than the untreated leached panels but were similar to other treated panels.

The mean heat release rate (HRR) varied significantly across the treatments. The untreated leached panels had the lowest mean HRR (97.8 ± 2.2 kW/m^2^), in contrast to the industrial reference non-leached panels, which had the highest HRR (112.2 ± 3.6 kW/m^2^), as well as the tannin-treated non-leached panels. The peak heat release rate was highest in the caffeine-treated non-leached panels (270.8 ± 8.3 kW/m^2^), differing significantly from both the industrial reference leached panels, which had the lowest peak HRR (210.4 ± 6.8 kW/m^2^), and the untreated leached panels.

Mass loss varied significantly among the panels, with the industrial reference leached panels showing the highest mass loss (82.3 ± 0.3%), notably different from the tannin-treated non-leached panels and the untreated non-leached panels. Total smoke production was greatest in the industrial reference non-leached panels (587.2 ± 22.2 m^2^/m^2^), significantly greater than the untreated leached panels, which produced the least smoke.

Time to flameout showed significant differences, with the industrial reference leached panels demonstrating the longest flameout time (872.8 ± 39.3s), notably longer than the tannin-treated non-leached panels. Total heat release (THR) was highest in the industrial reference leached panels (97.6 ± 2.7 MJ/m^2^) and significantly lower in untreated and tannin-treated non-leached panels.

## Discussion

The mass loss due to leaching of untreated OSB panel serves as a baseline for comparing the effectiveness of the treatments applied. Tannin-treated panels showed higher leaching than untreated panels, suggesting that tannins may have a greater tendency to leach out, possibly due to their solubility in water. This finding aligns with other studies highlighting the challenges in fixing tannins within the wood [34, 43]. In contrast, caffeine-treated panels exhibited lower mass loss due to leaching, similar to that of untreated panels and industrial references, indicating that caffeine interacts more effectively with the wood in this application. This contradicts previous studies, which reported that caffeine can leach out from solid wood when exposed to water [44]. One possible explanation for this discrepancy is that caffeine bonds to the phenol–formaldehyde resin. In addition, the panel manufacturing process, in which multiple strands are glued together, may hinder caffeine leaching compared to solid wood. Furthermore, the use of phenol–formaldehyde, which may act as a cross-linker, could further inhibit the leaching process [45].

The results of this study indicate that the treatments applied—caffeine and tannin—significantly reduced the mass loss due to decay caused by *T. versicolor*, *C. puteana*, and *G. trabeum* when compared to untreated and industrial reference panels. These findings have important implications for panel preservation, particularly in environments where fungal decay is a concern. Caffeine-treated panels demonstrated a substantial reduction in mass loss across all tested fungal species, indicating that caffeine effectively protects against fungal decay by bonding with phenol formaldehyde and thereby improving its performance. Previous studies have confirmed caffeine’s biocidal efficacy against wood-destroying fungi [32, 46].

Interestingly, the leached samples treated with caffeine also showed reduced decay, although the mass loss was slightly higher than that of the non-leached samples. This suggests that while caffeine is somewhat water-soluble and may leach out over time, it still retains enough efficacy to provide considerable protection, contrary to observation with solid wood, where caffeine has been reported to leach out [44]. The lower leachability of caffeine in the leached specimens may be attributed to the panel manufacturing process, where phenol formaldehyde plays a crucial role [47], and the strands are glued together, making leaching more difficult. This finding is important for applications where wood may be exposed to moisture, as caffeine treatment could still be effective under such conditions.

Tannin-treated panels also showed a significant reduction in mass loss. Tannins are recognized for their antifungal properties against wood-decaying fungi [31, 37, 48]. The results indicate that tannin treatment is particularly effective under non-leached conditions. While leached tannin-treated samples still showed reduced decay compared to untreated panels, they had higher mass loss percentages than their non-leached counterparts. This suggests that some of the protective effects of tannins may diminish with exposure to water, given their water-soluble nature. However, they still retain a degree of effectiveness, likely due to the panel manufacturing process, where strands are glued together, making leaching more difficult. Additionally, phenol–formaldehyde may act as a cross-linker, helping to reduce the leachability of tannins from the panels [47]. The strong performance of tannin-treated panels, especially under non-leached conditions, underscores the potential of tannin as a natural, environmentally friendly wood preservative.

The performance of non-leached tannin and caffeine treatments was comparable to that of commercial wood preservatives. Previous studies in wood preservation have documented the mass loss for various treatments, revealing that when exposed to *R. placenta*, micronized ACQ resulted in a 27.20 ± 6.09% mass loss, Nano CuO in a 19.27 ± 3.10% loss, CCA in a 4.10 ± 0.99% loss, and alkaline copper quaternary (ACQ) in a 3.09 ± 1.76% loss. Similarly, for *G. trabeum*, the mass loss was 20.21 ± 3.98% for Nano CuO, 12.99 ± 1.98% for ACQ, 4.23 ± 0.45% for micronized ACQ, and 3.21 ± 2.90% for CCA treatments [49]. In this context, the decay resistance observed for caffeine-treated OSB panels in the present study falls within the lower range of reported mass losses for conventional preservative systems.

In addition to preservative treatments, resin impregnation and wood modification approaches have been shown to significantly enhance decay resistance and, in some cases, mechanical performance. Phenol–formaldehyde resin impregnation, for instance, has demonstrated superior durability compared to some traditional preservative systems. However, such methods often rely on petrochemical-derived resins and energy-intensive processing, which may limit their sustainability advantages relative to naturally derived extractives. [50]. Furthermore, boric phenol formaldehyde resin (BPF) improved the mechanical properties and combustion performance of Chinese fir [51].

The comparable performance of the industrial reference and untreated panels highlights the potential benefits of alternative treatments, such as those tested in this study. Both caffeine and tannin treatments provided superior protection, particularly under non-leached conditions, suggesting they could be viable alternatives to OSB panel protection.

The significant reduction in fungal decay observed with caffeine and tannin treatments suggests that these compounds could serve as effective, natural alternatives to traditional wood preservatives. The efficacy of these treatments, particularly under non-leached conditions, supports their potential use in applications where wood is not continuously exposed to moisture, such as in indoor environments or structures with adequate weather protection. It should be emphasized that the present comparison is limited to laboratory decay resistance and does not imply equivalence in long-term outdoor performance or regulatory classification. However, the partial loss of efficacy in leached samples indicates that further research is needed to optimize these treatments for outdoor or highly humid conditions. In this context, it should be noted that, according to EN 113, fungal exposure is considered successfully passed when a mass loss below 3% is recorded after leaching.

The stronger retention of caffeine compared to tannin may be attributed to its smaller molecular size and higher mobility, which facilitate deeper penetration and more effective interaction with the phenol–formaldehyde matrix. In contrast, the larger and more heterogeneous tannin molecules are likely subject to steric hindrance, limiting the accessibility of reactive sites and reducing effective bonding. Furthermore, caffeine can form hydrogen bonds with the phenol–formaldehyde network, which likely contributes to its enhanced fixation, consistent with the observed lower leaching and improved decay resistance after leaching.

Future studies could explore combining caffeine and tannin with other hydrophobic treatments to enhance their resistance to leaching or investigate different application methods that could improve their durability.

Leached panels exhibited significantly longer times to ignition or no difference compared to their non-leached panels, which contrasts with previous studies that indicated leaching negatively affects fire properties [52]. This discrepancy may be due to the removal of soluble sugars and other wood compounds during the leaching process. Additionally, the tannin treatment did not differ from the untreated panels, contrary to previous studies where tannins were used as a fire retardant for wood. However, they were not as effective as commercial alternatives [53].

Non-leached panels had higher peak heat release rates, indicating that non-leached wood tends to release more energy during combustion due to its higher content of water-soluble compounds [54]. In contrast, the leached panels exhibited lower peak heat release rates, suggesting that leaching may remove chemical compounds contributing to increased peak heat release.

Future investigations should investigate the mechanisms underlying the potential fire-retardant effects of caffeine and tannin, including chemical or physical interactions with the resin, using techniques such as FTIR, SEM, or TGA.

The use of autoclave impregnation, a method widely adopted in industrial practices, underscores the feasibility of adapting this process for large-scale operations. Compared to traditional OSB panels, strand impregnation and additional drying would be required to remove excess water and ensure proper consolidation. As shown in previous work [55], these treatments do not compromise mechanical properties and may even enhance them in some cases. Nonetheless, achieving an optimal balance between the improved properties of the treated wood and economic efficiency will necessitate a comprehensive evaluation of cost factors and process optimization methodologies. Taken together, the present results suggest that the performance improvements observed could support the potential upscaling of this process, although further work is needed to fully assess industrial viability.

Current research is focused on identifying new natural alternatives for wood preservation. Several natural compounds and extracts, such as hydrothermal liquids [24] and monoterpenes [20], inhibit wood-decaying fungi. However, most of these proposed alternatives fail when applied in solid wood due to their high leachability. In contrast, the results obtained in the current study indicate that some natural preservatives, such as caffeine, may perform effectively in panels, even if they leach out from solid wood. This highlights the need for further studies of these natural preservatives to assess their potential application in panel manufacturing.

## Conclusion

This study demonstrates the potential of caffeine and tannins as effective preservatives for OSB panels. These treatments provided significant protection against decay caused *by T. versicolor, C. puteana, and G. trabeum*, outperforming both untreated panels and industrial reference samples, particularly under non-leached conditions. This suggests that caffeine and tannins could be viable alternatives to conventional preservatives for OSB. Notably, the leachability of caffeine and tannins from OSB panels was lower than that typically observed in solid wood. Further research is required to optimize these treatments for a broader range of environmental conditions, to evaluate their long-term durability, and to consider weathering tests, which would provide additional information on the potential use of this method in future applications.

## Data Availability

Data will be made available on reasonable request.
